# A diagnostic RNA sequencing assay for direct identification and interpretation of pathogenic variants in the *FBN1* gene

**DOI:** 10.3389/fmolb.2025.1693943

**Published:** 2025-11-03

**Authors:** Manal Irshaid, Ghadeera Al Mansoori, Mohamed Sulaiman, Asjed Mohamed, Mohammed Abdoh, Youssef M. Shalaby, Bashar Al-Zohily, Aalia Batool, Sara Aleissaee, Lara Alzyoud, Bassam R. Ali, Muna Al Saffar, Nadia Akawi

**Affiliations:** ^1^ Department of Genetics and Genomics, College of Medicine and Health Sciences, United Arab Emirates University, Al Ain, United Arab Emirates; ^2^ Department of Cardiology, Sheikh Shakhbout Medical City, Abu Dhabi, United Arab Emirates; ^3^ Department of Cardiology, KidsHeart Medical Center, Al Ain, United Arab Emirates; ^4^ Department of Pathology and Laboratories Medicine, Sheikh Shakhbout Medical City, Abu Dhabi, United Arab Emirates; ^5^ Division of Genetic and Genomics, Department of Pediatrics, Boston Children’s Hospital, Boston, MA, United States; ^6^ Division of Cardiovascular Medicine, University of Oxford, Oxford, United Kingdom

**Keywords:** Marfan syndrome, *FBN1*-mRNA sequencing, blood samples, RNA polymerase slippage, nested-polymerase chain reaction

## Abstract

**Introduction:**

The extensive size and multi-exon structure and the tissue-restricted expression of the associated gene *FBN1* challenge the genetic diagnosis of Marfan Syndrome (MFS). Current genetic diagnostic methods adopted clinically to confirm or rule out the disease diagnosis rely on high throughput DNA sequencing approaches, including whole exome or genome sequencing. While these approaches are powerful, they are costly, time-consuming, and labor-intensive, and they generate vast data sets that require computational and bioinformatic infrastructure to interpret. This study introduces an alternative sensitive, comprehensive, rapid, and cost-effective assay for genetic screening for MFS using whole blood RNA.

**Methods:**

Whole blood samples were collected in EDTA tubes, followed by immediate RNA and DNA extraction. A targeted RNA sequencing assay was designed to amplify and sequence the full coding region of FBN1 from whole blood, where Large overlapping cDNA fragments amplified from FBN1- RNA and directly sequenced, effectively addressing the challenge of low transcript expression utilizing nested PCR technique. The assay Applied to five unrelated families with suspected MFS enabled reliable detection of pathogenic variants identified by exome sequencing, and functional characterization of their transcriptional effects.

**Results:**

The assay identified four pathogenic FBN1 variants including one nonsense, two frameshift, and one missense, establishing the diagnosis in four cases. This corresponds to a diagnostic yield of 80%, exceeding that of whole exome sequencing, which identified variants in only three of the five families (60%).

**Conclusion:**

Beyond variant detection, the assay elucidates how these variants influence RNA transcription and contribute to pathogenic mechanisms. An insight that is often overlooked by DNA sequencing approaches. This allowed us to identify distinct effects of each identified variant and recognize RNA slippage as a novel disease mechanism that has not been reported before in MFS. The developed assay introduces an improved approach to the clinical genetic testing of MFS, with potential applicability for diagnosing other conditions involving large, multi-exon genes.

## Introduction

*FBN1* (fibrillin-1; MIM*134797) is a gene that encodes the large extracellular matrix glycoprotein fibrillin-1, a fundamental structural component of calcium-binding microfibrils (Handford, 2000). These microfibrils are critical in providing force-bearing structural support in elastic and non-elastic connective tissue throughout the body (Dietz, 2022). Pathogenic variants in *FBN1* are primarily linked to Marfan syndrome (MFS) and a broad spectrum of other autosomal dominant disorders, including MASS syndrome, ectopia lentis (EL), stiff skin syndrome, Weill–Marchesani syndrome, and acromicric dysplasia (Schrenk et al., 2018).

**FIGURE 1:**
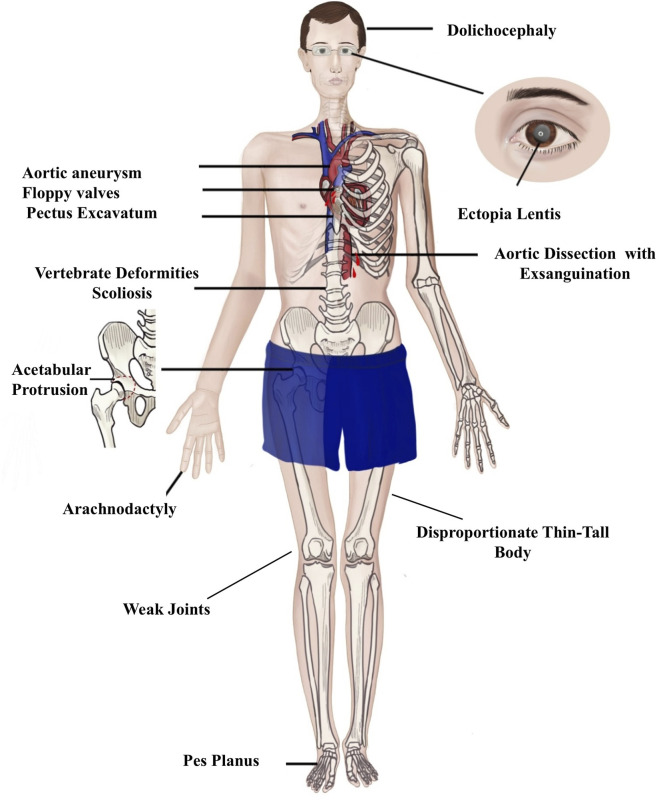
Clinical presentation of Marfan syndrome. The disease presents three primary manifestations: ocular, skeletal, and cardiovascular. Ocular involvement is the most prominent, characterized by ectopia lentis. Skeletal features primarily include disproportionately tall stature and scoliosis. The most severe complications are cardiovascular, with aortic dissection and aneurysms being the leading causes of mortality.

MFS exhibits a high degree of clinical variability, with a broad phenotypic spectrum characterized by three main clinical manifestations: ocular, skeletal, and, most significantly, cardiovascular (Figure 1) (Dietz, 2022). Although the classic clinical picture of MFS patients includes mainly a tall stature with disproportionately long limbs and fingers, chest wall deformities, scoliosis, joint hypermobility, and a high-arched palate, which are the hallmark signs of MFS (full article), these features are not unique to MFS and may overlap with other conditions such as Ehlers–Danlos syndrome, Loeys–Dietz syndrome, and homocystinuria (Meester et al., 2017; Verstraeten et al., 2016), making the clinical evaluation alone insufficient for a definitive disease diagnosis and, subsequently, clinical disease management.

Cardiovascular abnormalities, particularly aortic root dilation and dissection, EL, and systematic features characterized by disproportionately long stature, are key clinical diagnostic markers of MFS (Salik and Rawla, 2025). However, reliance on these features can result in missed diagnosis, especially in younger patients who may not show the aortic and ocular manifestations that typically worsen with age (Détaint et al., 2010; Sandvik et al., 2019) or in cases where a detailed family history is unavailable, as 25% of reported MFS cases arise from *de novo* pathogenic variants (Hu et al., 2022). This underscores the necessity of genetic testing as a fundamental tool for establishing the diagnosis. Clinically, the genetic testing approaches for MFS routinely utilize DNA as the primary genetic material for comprehensive genomic testing, mainly including exome sequencing (ES) and genome sequencing (GS), along with multi-gene panels (Dietz, 2022). Although these are powerful tools, they may overlook some non-coding variants in the causative gene. Although GS can reveal non-coding variants, their functional consequences are not always readily determinable. In addition, the approach is costly, time-consuming, and labor-intensive, and it generates vast datasets that require substantial computational and bioinformatics infrastructure for interpretation.

In this study, we developed a functional RNA sequencing assay to comprehensively screen the full-length canonical *FBN1* transcript (NM_000138.5) from whole-blood RNA, allowing sensitive detection of pathogenic *FBN1* variants and providing valuable insights into the mechanisms by which *FBN1* variants influence gene transcription and contribute to disease development. We introduce this assay as a diagnostic assay that can replace high-throughput techniques, providing an accurate diagnosis of *FBN1* pathogenic variants to confirm or rule out the disease.

## Patients and methods

### Patient recruitment and assessment

This study involved participants from five families with suspected MFS, who were recruited and evaluated at the Cardiac Genetic Clinic, Sheikh Shakhbout Medical City, Abu Dhabi, and The Heart Medical Center, Al Ain, United Arab Emirates. The study was approved by the ethical committee of the Department of Health of Abu Dhabi (DOH/CVDC/2023/1950).

The inclusion criteria mainly comprise patients clinically suspected of having MFS who exhibit specific marfanoid manifestations, particularly skeletal manifestations (tall stature, scoliosis, arachnodactyly, flat feet, and pectus excavatum), with or without cardiovascular manifestations (aortic dilation, aortic dissection, and mitral valve prolapse), ocular manifestations (ectopia lentis or myopia), or a family history of MFS.

### Exome sequencing

Blood samples were collected from the probands and the available family members in EDTA tubes. The plasma was separated, and DNA was extracted using the QIAamp DNA Mini Kit (QIAGEN, Hilden, Germany), following the manufacturer’s protocol. The quality and quantity of the extracted DNA were determined using NanoDrop spectrophotometer (Thermo Fisher Scientific, United States). The genomic DNA was fragmented to an average size of 180 bp–280 bp using an LE220-plus Focused-ultrasonicator (Covaris, Woburn, MA, United States). Library preparation and target enrichment were carried out using TruSeq DNA Exome (Illumina, United States). The libraries were sequenced with paired-end reads using S1 and SP flow cells on the NovaSeq 6000 platform (Illumina, San Diego, CA, United States).

A combination of the DRAGEN Bio-IT Platform (Illumina, United States) and in-house bioinformatics tools was used to read, map, align, and call variants. Variants were annotated and filtered using VarSeq 2.2.4 (Golden Helix, United States) and manual filtration. The only variants that passed quality-check filters (genotype quality >30; read depth >10) were further filtered based on minor allele frequency (<0.01 in the gnomAD database), and variants in the coding region (exons) and exon/intron boundaries ±20 bp, including missense, nonsense, frameshift, and splicing variants in disease-related genes, were investigated. The databases utilized to annotate and evaluate the impact of the detected variants are mainly gnomAD (https://gnomad.broadinstitute.org), HGMD (https://gnomad.broadinstitute.org), ClinVar (https://www.ncbi.nlm.nih.gov/clinvar), OMIM (https://www.omim.org), NCBI RefSeq (https://www.ncbi.nlm.nih.gov/refseq), dbSNP (https://www.ncbi.nlm.nih.gov/snp), PolyPhen-2 (http://genetics.bwh.harvard.edu/pph2), GERP++ (https://github.com/tvkent/GERPplusplus), MaxEntScan (http://hollywood.mit.edu/burgelab/maxent/Xmaxentscan_scoreseq.html), GeneSplicer (https://ccb.jhu.edu/software/genesplicer), NNSplice (https://www.fruitfly.org/seq_tools/splice.html), and SIFT ExAC Gene Constraints (https://sift.bii.a-star.edu.sg/). Variant classification was performed in accordance with the American College of Medical Genetics and Genomics (ACMG)/AMP guidelines, and interpretation was further guided by the recommendations of the ClinGen *FBN1* Variant Curation Expert Panel (VCEP).

The pathogenicity of the candidate variants was interpreted following the ACMG guidelines and considered all disease-causing variants reported in ClinVar (http://ncbi.nlm.nih.gov/clinvar; last accessed on 25 Dec 2024).

### Sanger sequencing

To validate the identified candidate variants and perform segregation analysis, genomic DNA from the proband and available family members was used to amplify the regions harboring the variants by standard polymerase chain reaction (PCR), using specific *FBN1* primer pairs designed with Primer3 (https://www.primer3plus.com/index.html) to flank the variants. Following PCR, Sanger sequencing was performed in both the forward and reverse directions via automated fluorescent sequencing on the ABI 3130xl Genetic Analyzer (Thermo Fisher Scientific, Waltham, MA, United States) using the BigDye Terminator v3.1 Cycle Sequencing Kit (Applied Biosystems, United States). Sequencing data were analyzed using multiple sequence alignment tools, including BioEdit software and Clustal Omega.

### *FBN1-*RNA sequencing

To study the impact of the detected variants on *FBN1*-mRNA, mRNA was extracted from blood samples collected in EDTA tubes directly after blood collection using the QIAamp RNA Mini Kit (QIAGEN, Hilden, Germany), following the manufacturer’s recommendations, and the protocol was optimized in our laboratory to improve the yield and purity. The concentration and purity of the extracted RNA were checked using a NanoDrop spectrophotometer (Thermo Fisher Scientific, United States). Total RNA (0.5 ug) was used as a template for cDNA synthesis using the GoScript™ Reverse Transcription Kit (Promega, Madison, WI, United States). The *FBN1* consensus coding sequence was retrieved from NCBI (https://www.ncbi.nlm.nih.gov/). The complete *FBN1*-mRNA was amplified through two rounds of PCR, comprising first and nested PCR cycles. Primers for *FBN1*-mRNA were designed using Primer3 (www.primer3.sourceforge.net), and successful amplification was confirmed using 1.5% agarose gel containing ethidium bromide. Nested PCR products were sequenced directly in both directions using the BigDye Terminator v3.1 Cycle Sequencing Kit (Applied Biosystems, United States) via automated fluorescent sequencing on the ABI 3130xl Genetic Analyzer (Thermo Fisher Scientific, Waltham, MA, United States), and sequencing data were analyzed using BioEdit and Clustal Omega software. The assay was validated on a control sample obtained from a healthy donor before being applied to the patient’s samples.

### Quantification of relative *FBN1* gene expression

Relative *FBN1* gene expression was measured via quantitative real-time PCR (qPCR system) on the QuantStudio™ 7 Flex Real-Time PCR System (Thermo Fisher Scientific, United States). cDNA was synthesized via RT-PCR, and 500 ng of cDNA was synthesized from each RNA sample using the GoScript Reverse Transcription System (Promega, Wisconsin, United States, Cat. No A5001). In brief, 0.5 µL of a random primer was added to the RNA, brought to a total volume of 5 µL with nuclease-free water, heated at 70 °C for 5 min, and then chilled on ice. The reverse transcription mix was prepared by combining GoScript 5X reaction buffer, MgCl_2_ (1.5 mM–5.0 mM), dNTPs (0.5 mM each), RNasin ribonuclease inhibitor (20 units), reverse transcriptase (1 µL), and nuclease-free water to a final volume of 15 µL. This mix was added to the RNA–primer solution, and reverse transcription was performed on a thermal cycler under the following conditions: 25 °C for 5 min, 42 °C for 1 h, and, finally, 70 °C for 15 min.

Relative gene expression analysis utilized the TaqMan Gene Expression Assay for *FBN1* (Hs00171191-m1, Applied Biosystems) and glyceraldehyde-3-phosphate dehydrogenase (*GAPDH*) (Hs99999905_m1, Applied Biosystems). Reactions were carried out in duplicates in 96-well plates for the affected patients (n = 13) and healthy controls (n = 13) with no known connective tissue disorders, matched for age and gender to the affected patients. Water served as a negative control. The levels of *FBN1* were normalized to the levels of *GAPDH* and calculated following the 2^−ddCT^ method.

### Quantification of fibrillin-1 levels in plasma

A solid-phase sandwich enzyme-linked immunosorbent assay was used to measure the levels of fibrillin-1 in the plasma of affected patients (n = 13) and age- and gender-matched healthy controls (n = 13) with no known connective tissue disorders. Reactions were performed in duplicate using the Human Fibrillin-1 ELISA Kit (Cat#EEL134; Invitrogen, New York, United States), following the manufacturer’s protocol, and the optical density was measured at 450 nm using a Biotech Synergy H1 Microplate Reader (Agilent Technologies, California, United States). Fibrillin-1 concentration was measured using the following equation: (fibrillin-1 (ng/mL) = (OD sample − OD blank)/slope of the standard curve, where OD is the optical density, and the slope of the standard curve was obtained from the linear regression of fibrillin-1 standards.

### Statistical analysis

Relative *FBN1* gene expression and plasma fibrillin-1 concentrations in the affected patients and control samples were analyzed using GraphPad Prism (version 10.2.2, Boston, Massachusetts, United States). The data were tested for normality using the Shapiro–Wilk test, and outliers were excluded using Grubbs’ test. The Mann–Whitney U test was used for analyzing non-normally distributed data, while normally distributed data were analyzed using an unpaired t-test. All data were expressed as the mean ± SEM, and *p* < 0.05 was considered statistically significant.

## Results

### Marfanoid features segregation across five families

**FIGURE 2:**
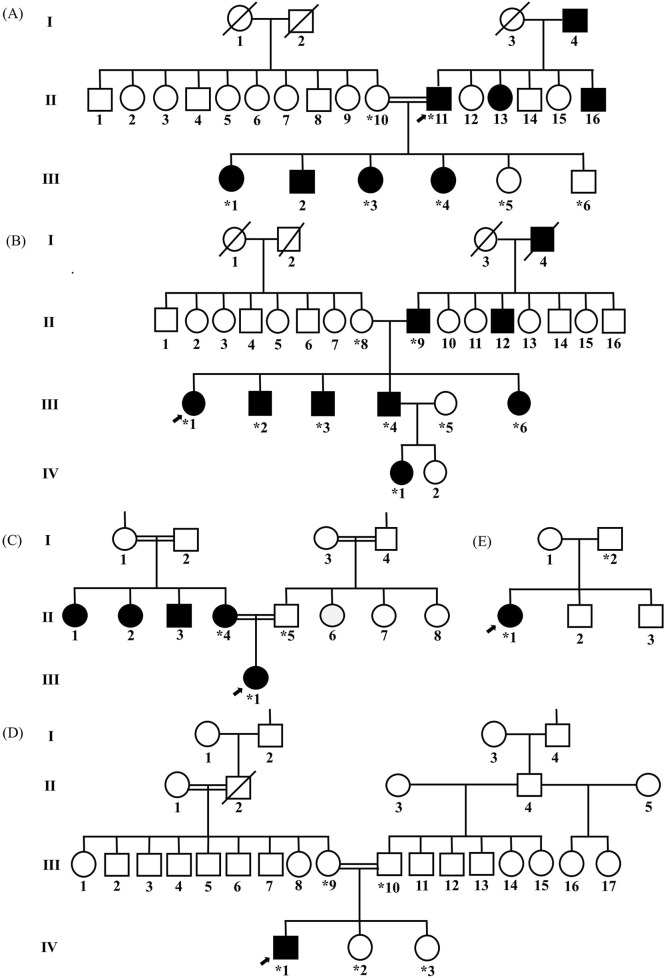
Pedigree analysis of five families, highlighting cardiovascular and skeletal manifestations associated with marfanoid features. In family 1, the proband (II:11) exhibited a remarkable tall stature and severe cardiac manifestations, primarily aortic root dilation **(A)**. These abnormalities were observed in some of his offspring in generation III, including III:1, III:2, III:3, and III:4. All displayed tall stature, exceeding the 95th percentile for their respective gender and age groups. In family 2, clinical assessment of the proband (III:1) revealed multiple marfanoid features, predominantly including cardiac complications, with severe aortic root dilation and aneurysm, and skeletal abnormalities, mainly scoliosis **(B)**. These features were variably present among other family members, including II:9, III:2, III:3, III:4, III:6, and IV:1. In family 3, the proband (III:1) exhibited mild cardiac phenotypes, including mitral and tricuspid valve prolapse, trivial mitral regurgitation, and tricuspid regurgitation **(C)**. Additionally, scoliosis was noted as a skeletal abnormality, which was also identified in her mother (II:4). In family 4, the proband presented with skeletal anomalies, including scoliosis and joint laxity, without any ocular or cardiac involvement **(D)**. No other family members exhibited similar findings. In family 5, the proband (II:1) exhibited a tall stature (above the 95th percentile for gender and age) and cardiac abnormalities, including an atrial septal defect with a left-to-right shunt, trace mitral regurgitation, and mild pulmonary and tricuspid valve regurgitation. However, other family members did not show these cardiac findings **(E)**. The arrow indicates the proband of each family. Circles represent female participants, squares represent male participants, open samples represent unaffected subjects, solid symbols indicate affected patients, the slash through the symbols indicates deceased subjects, and the vertical line above the square or circle represents a distal relationship. The Roman numbers (I, II, III, and IV) represent the generations, while the other numbers (1, 2, 3…) represent the specific individual in each generation, and * indicates the individuals who are genetically tested.

This study recruited five probands with variable marfanoid features and available family members. The pedigrees and the clinical manifestations of the recruited individuals are detailed in Figures 2A–E and Table 1.

**TABLE 1:** Clinical findings of the probands and the available family members.

ID	Gender	Age of diagnosis	Cardiac manifestations	Ocular manifestations	Skeletal manifestations	Surgical intervention	Comorbidities
Family 1							
II:10	Female	46 years	-	-	-	-	Hypertension, Lipidaemia, History of breast cancer
II:11	Male	49 years	LVH, Aortic root dilation, AR, MVP, MR	-	Hight: 182 cm (above 95%)	Bentall procedure	Hypertension
III:1	Female	25 years	LV concentric remodeling, Aortic root dilation, AR, Bileaflet MVP, MR	-	Hight: 177 cm (above 95%)	Aortic root replacement, Bentall procedure	-
III:2	Male	23 years	Aortic root dilation, AR, PFO, LVH and concentric remodeling, Decreased systolic function, Hypokinesia, Interventricular septal desynchrony, LA dilation, AR, Pericardial effusion	-	Hight: 177 cm (above 95%)	David procedure, PFO closure	-
III:3	Female	17 years	MR, MVP, pericardial effusion, TR,	-	170 cm (above 95%)	-	-
III:4	Female	19 years	AF, RBBB,	-	Hight: 173 cm (above 95%)	-	-
III:5	Female	14 years	-	-	-	-	-
III:6	Male	21 years	-	-	-	-	-
Family 2							
II:8	Female	62 years	-	Hyperopia	Osteoarthritis	-	DM
II:9	Male	63 years	Aortic root dilation	EL	Hight: 182 (above 95%) Joint laxity	CABG Lense implantation,	Renal Disease, DM, GERD, PVD, BKA
III:1	Female	32 years	Aortic root aneurysm, Ascending Aorta root dilation (50 mm)	EL	Hight: 183 cm (above 95%), Cervical and several intervertebral discs degeneration, joint laxity	CABG, Lense implantation	Arachnoid cyst, Hypertension, Hyperlipidemia
III:2	Male	22 years	Aortic root aneurysm, increased sinotubular junction,	EL	Hight: 199 cm (above 95%) hyperextensible joints, Pectus Excavactum, Overcrowded teeth, Joint laxity	Lense implantation, Bentall procedure	Hypertension
III:3	Male	27 years	Aortic root aneurysm	Myopia	Hight: 195 cm (above 95%), Overcrowded teeth	-	soft tissue nodules attached to the anterior inferior articular wall, hepatic lesion in segment VIII of the liver
III:4	Male	30 years	Aortic root dilation	EL	Hight: 195 cm (above 95%), Joint Laxity	Lense implantation, Bentall procedure	-
III:5	Female	28 years	-	-	-	-	-
III:6	Female	33 years	-	Myopia	Scoliosis Hight: 183 cm (above 95%)	-	-
IV:1	Female	7 years	-	Myopia	-	-	-
IV:2	Female	2 months	-	-	-	-	-
Family 3							
II:4	Female	32 years	-	Myopia	Scoliosis Hight: 178 cm (above 95%)	-	Family history of breast cancer, Leukemia,
II:5	Male	36 years	-	-	-		-
III:1	Female	12 years	Aortic root dilation, MVP, TR aortic root dilation	EL	Scoliosis, Hight: 170 cm (above 95%) advanced bone age, disproportionately tall stature	-	Product of IVF pregnancy, precocious puberty,
Family 4							
III:9	Female	45 years	-	-	-	-	-
III:10	Male	47 years	-				-
IV:1	Male	17 years	loud S2 and an ejection systolic murmur at the left sternal border	-	Hyper joints laxity, increase in skin elasticity, Hight: 177 cm	-	-
IV:2	Female	12 years	-	-	-	-	-
IV:3	Female	10 years	-	-	-	-	-
Family 5							
I:1	Female	38	-	-	-	-	-
I:2	Male	40	-	-	-	-	-
II:1	Female	13	ASD with left to right shunt, Mild MR, Mild pulmonary and tricuspid valve regurgitation		Hight: 168 (above 95%)	-	Intellectual impairment
II:2	Male	7	-	-	-	-	-
II:3	Male	3	-	-	-	-	-

LV, Left Ventricular; LVH, Left Ventricular Hypertrophy; AA, Ascending Aorta; AR, Aortic Regurgitation; MVP, Mitral Valve Prolapse; MR, Mitral Regurgitation; PFO, Patent Foramen Ovale; LA, Left Atrium; TR, Tricuspid Regurgitation; AF, Atrial Fibrillation; RBBB, Right Bundle Branch Block; ASD, Atrial Septal Defect; DM, Diabetes mellitus; GERD, Gastroesophageal Reflux Disease; PVD, peripheral vascular disease; BKA, Below-knee Amputation; CABG, Coronary Artery Bypass Graft; EL, Ectopia lentis.

#### Family 1

A 49-year-old Emirati male (II:11; Figure 2A) was referred to the cardiology clinic due to a history of hypertension. The echocardiographic evaluation revealed severe dilation of the aortic root and ascending aorta, left ventricular concentric hypertrophy, mild-to-moderate aortic regurgitation, and mitral regurgitation. Cardiac abnormalities were further identified in four family members (III:1, III:2, III:3, and III:4), with the most common pathology being aortic root dilation, followed by mitral valve disease, which included mitral valve prolapse (MVP) and regurgitation. Five individuals (II:11, III:1, III:2, III:3, and III:4) had heights exceeding the 95th percentile of their related age and gender group. Elective aortic root replacement was performed in three individuals (II:11, III:1, and III:2). Additionally, III:2 underwent surgical closure of a patent foramen ovale, which was complicated by acute postoperative pericarditis 4 days after the procedure.

#### Family 2

A 32-year-old Palestinian female (III:1; Figure 2B) showed redundant mitral valve leaflets with anterior leaflet prolapse, eccentric mitral regurgitation, and aortic root aneurysm with significant dilation. Subsequent cardiac assessments of additional family members revealed various cardiac abnormalities in II:9, III:2, III:3, and III:4, with aortic root dilation and aneurysm as the predominant findings. Several family members had undergone cardiac surgeries, including a coronary artery bypass graft (CABG; III:9 and III:1) and Bentall procedure (III:2 and III:4). Ocular manifestations, including EL and myopia, were observed in some individuals (II:9, III:1, III:2, III:3, III:4, III:6, and IV:1), where four of them had undergone lens implantation (II:9, III:1, III:2, and III:4). The family members exhibited various skeletal manifestations, as described in Table 1, and six of them (II:9, III:1, III:2, III:3, III:4, and III:6) exhibited stature exceeding the 95th percentile for their respective age and gender groups.

#### Family 3

A 12-year-old Omani girl (III:1; Figure 2C), an IVF-conceived daughter of consanguineous Omani parents, was referred for cardiac evaluation. An echocardiogram indicated the presence of MVP, accompanied by trivial mitral regurgitation and mild tricuspid valve prolapse with mild tricuspid regurgitation. The skeletal assessment revealed scoliosis with a Cobb angle measurement of 40°, in addition to disproportionately tall stature, with a height of 170 cm, placing her above the 95th percentile for her gender and age group. The ocular examination demonstrated mild upward lens subluxation. Physical examination of the parents identified mild scoliosis in the mother (II:4), who was 178 cm tall, placing her and her siblings II:1, II:2, and II:3, who were 177 cm–180 cm tall, above the 95th percentile for their age and gender groups. No cardiovascular manifestations were identified in any of them.

#### Family 4

A 17-year-old Comorian male (IV:1; Figure 2D) was referred for evaluation as a suspected case of MFS. The proband had no reported cardiovascular symptoms and reported no family history of cardiovascular conditions such as aortic dilation or aortopathy, suggesting no immediate hereditary concerns. The patient was 177 cm tall, with signs of joint laxity, and had no chest wall deformity or scoliosis; his ocular examination was unremarkable. Cardiovascular examination demonstrated a regular heart rate and rhythm. Despite normal ECG findings with no evidence of structural or functional heart abnormalities, a 48-h Holter monitor revealed periods of sinus bradycardia and tachycardia, with a minimum heart rate of 44 bpm, a maximum of 176 bpm, and an average of 80 bpm. A stress test was also performed, and the results were within normal limits, indicating no inducible ischemia or arrhythmia under physical exertion.

#### Family 5

A 13-year-old Egyptian female was referred for cardiac evaluation secondary to an incidental murmur. The electrocardiogram revealed sinus arrhythmia, and the echocardiogram showed a tiny atrial septal defect with a left-to-right shunt, trace mitral regurgitation, and mild pulmonary and tricuspid valve regurgitation; the proband had no documented family history of cardiovascular disease. The girl was 163 cm tall (placing her above the 95th percentile for her age and sex) and exhibited no ocular abnormalities.

### DNA sequencing reveals pathogenic *FBN1* variants in three out of five studied families

**FIGURE 3:**
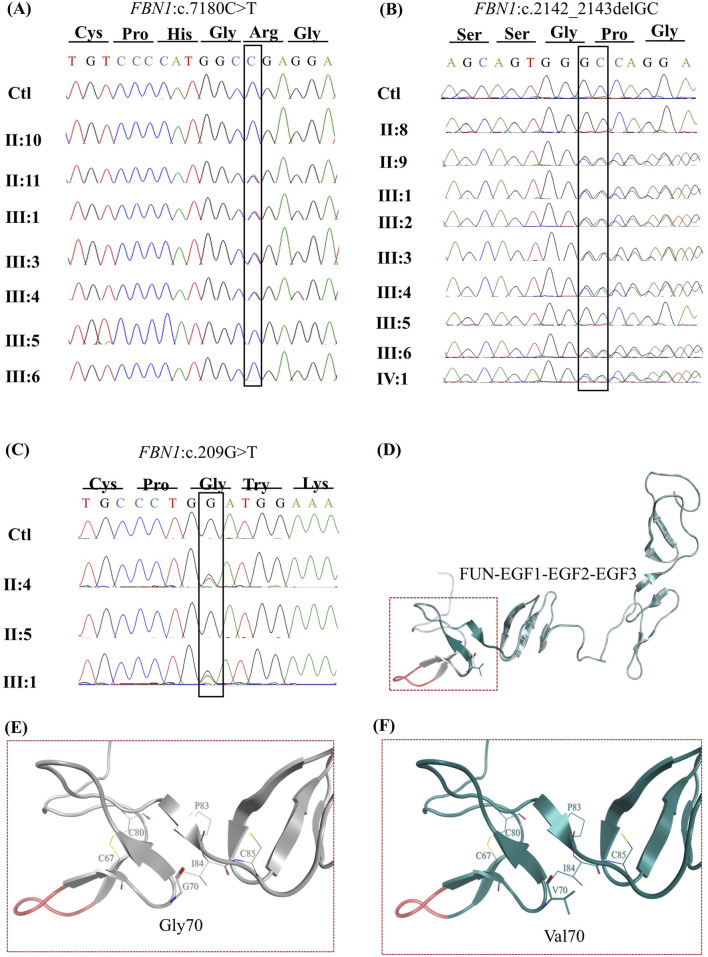
Inheritance patterns and structural impact of *FBN1* variants identified through exome sequencing. Sanger sequencing chromatograms demonstrate the transmission of the FBN1:c.7180C>T variant in a heterozygous state (double peaks) from the father II:11 to his children III:1, III:3, and III:4 **(A)**. Sanger sequencing chromatograms illustrating the inheritance of the (NM_000138.5(FBN1):c.2142_2143del (p.Pro715Argfs*8)) variant in a heterozygous state (evidenced by peak shifts at the mutation site) from the father II:9 to all his children III:1, III:2, III:3, III:4, III:6, and IV:5 **(B)**. Sanger sequencing chromatograms showing the transmission of the FBN1:c.209G>T variant in a heterozygous state (double peaks) from the mother II:4 to her daughter III:1 **(C)**. The FUN-EGF1–EGF2–EGF3 domains of the fibrillin-1 protein (PDB: 2M74) **(D)**. Structural representation of the Gly70Val mutation in the fibrillin-1 protein, preserving the disulfide bond between CYS67 and CYS80 in wild-type **(E)** and mutant forms **(F)**.

ES of the five probands of this study detected 35 variants in the *FBN1* gene, most of which are non-coding (Supplementary Table S1). Three potentially causal variants were identified and confirmed using Sanger sequencing (Supplementary Table S2), including a heterozygous pathogenic nonsense variant (NM_000138.5(*FBN1*): c.7180C>T p.(Arg2394*)) in the proband of family 1 that segregated with the phenotype from II:11 to his offspring III:1, III:3, and III:4 (Figure 3A). The variant is rare in gnomAD and has been reported in ClinVar and the literature as pathogenic in several MFS cases (Zhurayev et al., 2016; Whiteman and Handford, 2003) (Yang et al., 2016; Yoo et al., 2010; Drackley et al., 2024).

A likely pathogenic frameshift variant (NM_000138.5*(FBN1)*:c.2142_2143del (p.Pro715Argfs*8)) was identified in family 2 and segregated from the father II:9 to his children III:1, III:2, III:3, III:4, and III:6 and his grandchild IV:1 (Figure 3B). The variant is novel and absent from gnomAD, ClinVar, and the clinical literature.

A likely pathogenic missense variant (NM_000138.5(*FBN1*):c.209G>T p.(Gly70Val)) was detected in family 3 and segregated with the phenotype from the mother II:4 to her daughter III:1 (Figure 3C). The variant changed glycine (Gly) at position 70 into valine (Val). Gly is highly conserved in available vertebrate species (phyloP100 = 7.299) and predicted to be deleterious (SIFT score = 0.00, CADD = 32). The variant was reported in an MFS patient (Guo et al., 2015), and it is not reported in the gnomAD database or the literature.

Gly70 is part of the fibrillin unique N-terminal (FUN) domain, containing a disulfide bond domain involved in the formation of a disulfide bond (Cys67–Cys80) (FBN1), which plays a significant role in the protein assembly by interacting with the C-terminal domain of the protein, suggesting that replacement of the amino acid at this position with a different amino acid can impact the protein’s folding and, subsequently, the protein’s function (Figure 3D). However, computational 3D modeling of the FUN domain harboring the mutated amino acid presents a preserved disulfide bond (Figures 3E, F).

No pathogenic variants were identified in the *FBN1* gene in families 4 and 5. Variants of uncertain significance were identified in other genes associated with marfanoid features (Supplementary Table S2). All the above-mentioned variants are extremely rare in the Emirati Reference Genome (ERG) datasets and are not found in the in-house cohort of 840 exomes.

### RNA sequencing uncovers an additional pathogenic variant and highlights the impact of detected DNA variants

**FIGURE 4:**
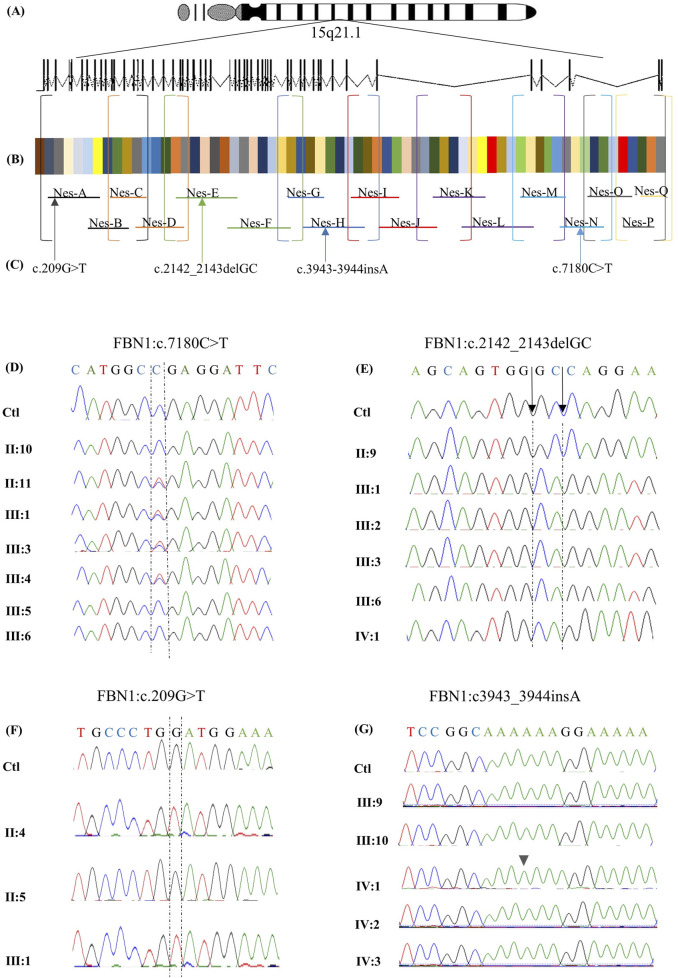
Targeted RNA-sequencing assay design for *FBN1* gene analysis. A targeted RNA-sequencing assay was designed to analyze the *FBN1* gene on chromosome 15q21.1, spanning 66 exons **(A)**. The two-step RT-PCR method was optimized to amplify the 8,616-nucleotide coding sequence of the canonical *FBN1* transcript (NM_000138.5) **(B)**. Overlapping primers (P1 and P2) were developed to ensure full coding sequence coverage. In the first round of PCR (P1), 9 cDNA fragments were generated and amplified through 17 nested PCR reactions (P2). The final products were sequenced using Sanger sequencing, which revealed multiple pathogenic variants **(C)**. The identified variants are aligned to the corresponding exon, and the primer amplifies the RNA segment hosting it. Variant c.209G>T in exon 3 is amplified by primer A, variant NM_000138.5(FBN1):c.2142_2143del (p.Pro715Argfs*8) located in exon 18 is amplified by primer E, variant NM_000138.5(FBN1):c.3945dup (p.Gly1316Argfs*10) in exon 32 is amplified by primer H, and variant c.7180C>T in exon 58 is amplified by primer N. Chromatograms obtained by Sanger sequencing of RNA from available members of family 1 **(D)**. The healthy members, including II:10, III:5, and III:6, expressed the wild-type allele. In contrast, the affected family members carrying the nonsense variant c.7180C>T, including II:11, III:1, III:3, and III:4, expressed both the wild-type and the mutated alleles chromatograms obtained by Sanger sequencing of RNA from the available members of family 2 **(E)**. The chromatograms show that the healthy member (II:9) expressed the wild-type allele, while the affected family members carrying the frameshift variant NM_000138.5(FBN1):c.2142_2143del (p.Pro715Argfs*8) (including III:1, III:2, III:3, III:6, and IV:1) exclusively expressed the mutated allele. Chromatograms obtained by Sanger sequencing of RNA from the available members of family 3 **(F)**. The chromatograms show that the healthy member (II:5) expressed the wild-type allele. In contrast, the affected family members carrying the missense variant c.209G>T, including II:4 and III:1, exclusively expressed only the mutated allele. Sanger sequencing chromatograms of RNA from the available family members indicate no RNA abnormalities, except for the proband IV:1, who exhibited the NM_000138.5(FBN1):c.3945dup (p.Gly1316Argfs*10) variant, which was absent at the DNA level **(G)**.

To evaluate the impact of coding and non-coding variants on the *FBN1* (NM_000138.5) transcript, a targeted RNA sequencing assay was developed to amplify 8,616 bases through two rounds of RT-PCR (Figure 4A). The first round amplifies nine distinct cDNA fragments, which are subsequently subjected to a second round of 17 reactions, each nested within the 9 initial reactions (Table 2; Figure 4B; Supplementary Figure S1).

**TABLE 2:** Primers used for *FBN1*-mRNA amplification and the optimized RT-PCR conditions.

Primer	Forward primer	Reverse primer	Exons amplified	Region amplified	Product size (bp)	Initial denaturation	Denaturation	Annealing	Extension	Final extension	Number of cycles
FBN1-1	CCTGAAGTGGG AGCAGCG	AGTATCCTGGGC GAACATCT	Exon 2-10	-168-1006	1174						
Nes-A	CCCTGGGATTT ACCGTGCTT	GCAGAGCGTT TTTGTGCAGA	Exon 2-7	29-627	599	95°C/ 5 minutes	95°C/ 45 seconds	56°C/ 45 seconds	72°C/ 45 seconds	72°C/ 7 minutes	35
Nes-B	GGTAGCTGC AGTGACGATCA	TCCAGGAATG GTGCTGCATT	Exon 5-9	379-885	507						
FBN1-2	TCTGTCAGGG AGGAAATTGCA	CTCCGCATG TGTGTGTCAAC	Exon 8-16	767-1976	1210						
Nes-C	CTGTTGGGTC TTTTGAGTGCA	GCGTCCATTT TGACAGAGATAGC	Exon 8-12	794-1392	599	95°C/ 5 minutes	95°C/ 45 seconds	56°C/ 45 seconds	72°C/ 45 seconds	72°C/ 7 minutes	35
Nes D	TGTATCCATCTC GGGAGCCA	CATGCAGATC CCAGGGGT	Exon 11-16	1304-1872	569						
FBN1-3	CGTGTGTAATG CGGGCTTTC	GAGGTCAGGAG ATATGCGGC	Exon 14-27	1665-3240	1576						
Nes-E	TGTGTATCAAT GAAGATGGCAGT	AGGACTTGATT CGCATTCATCA	Exon 15-21	1757-2446	690	95°C/ 5 minutes	95°C/ 45 seconds	58°C/ 45 seconds	72°C/ 45 seconds	72°C/ 7 minutes	35
Nes-F	CTCCTGGAAGTT TTGTCTGTACC	CGCTGTCACACCT GCACTTA	Exon 20-26	2351-3172	822						
FBN1-4	CTGGGGTACT GAGGAATGCG	AGGGAGGTTG TGGCAAGTTC	Exon 26-35	2961-4395	1435						
Nes-G	GCAAGATGATA CCCAGCCTCTG	CACAAGCACCTG TACTCTCCA	Exon 27-30	3095-3794	700	95°C/ 5 minutes	95°C/ 45 seconds	58°C/ 45 seconds	72°C/ 45 seconds	72°C/ 7 minutes	35
Nes-H	TGGCTACCATT CAACTCCCG	TGCCACAGAGA TTCAGGTTCT	Exon 28-35	3552-4249	698						
FBN1-5	TGAAATTGGAG CACACAACTGT	CACACTGGGC CGTTCTGAC	Exon 33-45	3978-5453	1476						
Nes-I	GGGTGGATTGG AGATGGCAT	CCAGAGAACAG CAGCAGGAA	Exon 33-38	4057-4699	643	95°C/ 5 minutes	95°C/ 45 seconds	58°C/ 45 seconds	72°C/ 45 seconds	72°C/ 7 minutes	35
Nes-J	GCTGTGTTG ATACCCGCTCT	AGCAGCACATCT TCTTGGTCA	Exon 36-42	4574-5161	588						
FBN1-6	TCCAACCGGCT ACTACCTGA	TTCACACATCGG AAGGCACA	Exon 40-55	4899-6668	1770						
Nes-K	TGCTACAGAAAC TACTATGCTGACA	TGTTGTGAGAAA GGATGAAACCA	Exon 42-47	5083-5776	694	95°C/ 5 minutes	95°C/ 45 seconds	58°C/ 45 seconds	72°C/ 45 seconds	72°C/ 7 minutes	35
Nes-L	CTACCGCTTCACC TCCACAG	CCCACGATGATC CCACTTCC	Exon 45-52	5514-6359	846						
FBN1-7	GAAGGTGCCAA G ATTTGCGA	TTTCGGGCATGA ACACTGGT	Exon 50-60	6152 -7416	1265						
Nes-M	GCCCCACGGAA CCTGATG	CTCATCTACAC AGCCTTCTCCA	Exon 51-56	6296-6876	580	95°C/ 5 minutes	95°C/ 45 seconds	58°C/ 45 seconds	72°C/ 45 seconds	72°C/ 7 minutes	35
Nes-N	ACAAATGGAAT GCAAGAACCTCA	GGAGCCTGGT TGCACTCG	Exon 56-60	6783-7355	573						
FBN1-8	ACAATCGGGAA GGGTACTGC	CGTCAGAGTTG TAAGAGCTGGA	Exon 58-66	6998-8364	1367	95°C/ 5 minutes	95°C/ 45 seconds	58°C/ 45 seconds	72°C/ 45 seconds	72°C/ 7 minutes	35
Nes-O	ACTGTGAGATC TGCCCTTTCC	AACACACTGGT TCCACTGGT	Exon 58-63	7121-7818	698						
Nes-P	CAGCGGGGAT TCTCACTTGA	GGCATCAGTTT CGTTTGTGCT	Exon 62-65	7657-8214	558						
FBN1-9	CCAACACCATA CGTCCTGCA	GAAGCCTGAG AAAGTGGTTGT	Exon 61-66	7548-+199	1264	95°C/ 5 minutes	95°C/ 45 seconds	56°C/ 45 seconds	72°C/ 45 seconds	72°C/ 7 minutes	35
Nes-Q	CTCCTGTCACA ACACCCTGG	TTATTTGGTC TCTGGATGGTGAA	Exon 64-66	7860-+23	780						

Sequencing of RNA derived from the proband carrying the nonsense variant c.7180C>T revealed the presence of two peaks at position 7,180, representing the wild-type (WT) and the mutated alleles (Figures 4C, D), making the mutated transcript available to produce a truncated protein that is 477 amino acids shorter than the WT. Similar results were obtained for all affected members of family 1. In family 2, the expression of only the mutated transcript was detected in all the affected members (Figures 4C, E), making the mutated transcript the only available transcript to produce a truncated protein that contains only 723 amino acids, which is 2,148 amino acids shorter than the WT comprising 2,871 amino acids. Similarly, RNA sequencing of both the affected members in family 3 revealed the exclusive expression of the mutated allele, while the WT allele was not expressed (Figures 4C, F).

In family 4, *FBN1*-mRNA was normal in all the family members except for the proband. RNA sequencing revealed the presence of a damaging variant (NM_000138.5(*FBN1*):c.3945dup (p.Gly1316Argfs*10)) (Figures 4C, G), causing a frameshift change in exon 32, resulting in an alternate stop codon (p.G1316Rfs*10). The variant was not detected on the DNA level, suggesting that it resulted from RNA-polymerase slippage on repetitive sequences where the variant is located (AAAAAAGGAAAAA). The variant was absent from gnomAD. The variant has been reported in ClinVar as a pathogenic variant. This variant was not detected in the parents, other family members, or the other investigated individuals (Supplementary Figure S2).

In family 5, no apparent abnormalities in *FBN1*-mRNA were detected in the proband or any other family members. No other mis-splicing events were identified in the RNA of all the affected members of the five families, ruling out the possible effects of all other identified intronic variants on RNA transcription. The results of both DNA and RNA sequencing of the recruited members of the five families are described in Table 3.

**TABLE 3:** Summary of the sequencing results for all the recruited members in the five families.

ID	Candidate exome sequencing result	Sanger sequencing result	*FBN1*-mRNA sequencing result	
			Variant	Allele
Family 1				
II:10	-	Negative	Negative	WT
II:11	*FBN1*: c.7180C>T	*FBN1*: c.7180C>T	*FBN1*: c.7180C>T	WT and mutant
III:1	-	*FBN1*: c.7180C>T	*FBN1*: c.7180C>T	WT and mutant
III:3	-	FBN1: c.7180C>T	*FBN1*: c.7180C>T	WT and mutant
III:4	-	*FBN1*: c.7180C>T	*FBN1*: c.7180C>T	WT and mutant
III:5	-	Negative	Negative	WT
III:6	-	Negative	Negative	WT
Family 2				
II:8	-	Negative	Negative	WT
II:9	-	*FBN1*: c.2142_2143delGC	*FBN1*: c.2142_2143delGC	-
III:1	NM_000138.5(FBN1):c.2142_2143del (p.Pro715Argfs*8)	*FBN1*: c.2142_2143delGC	*FBN1*: c.2142_2143delGC	Mutant
III:2	-	*FBN1*: c.2142_2143delGC	*FBN1*: c.2142_2143delGC	Mutant
III:3	-	*FBN1*: c.2142_2143delGC	*FBN1*: c.2142_2143delGC	Mutant
III:4	-	*FBN1*: c.2142_2143delGC	*FBN1*: c.2142_2143delGC	-
III:5	-	Negative	Negative	-
III:6	-	*FBN1*: c.2142_2143delGC	*FBN1*: c.2142_2143delGC	Mutant
IV:1	-	*FBN1*: c.2142_2143delGC	*FBN1*: c.2142_2143delGC	Mutant
Family 3				
II:4	-	*FBN1*: c.209G>T	*FBN1*: c.209G>T	Mutant
II:5	-	Negative	Negative	WT
III:1	-	*FBN1*: c.209G>T	*FBN1*: c.209G>T	Mutant
Family 4				
III:9	-	-	Negative	WT
III:10	-	-	Negative	WT
IV:1	*Negative*	-	NM_000138.5(FBN1): c.3945dup (p.Gly1316Argfs*10)	Mutant
IV:2	-	-	Negative	WT
IV:3	-	-	Negative	WT
Family 5				
5/I:2	-	-	Negative	WT
5/II:1	*Negative*		Negative	WT
5/II:2	-	-	Negative	WT

(−), not performed; WT, wild-type allele.

### Quantitative assessment of variants’ effects on *FBN1* gene expression

**FIGURE 5:**
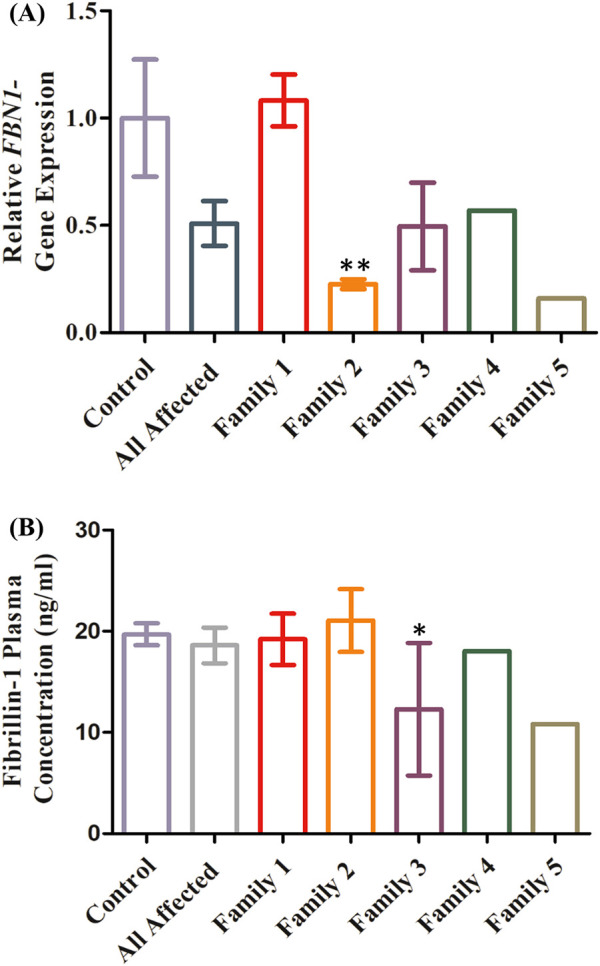
Quantitative assessment of the identified variants. A comparison of *FBN1* expression between the controls and all the affected members from the five families shows that the expression decreased by 50% compared to that in the control **(A)**. A comparison of *FBN1* expression between the controls and affected members from family 1 indicated almost equal expression with a slight increase of 8%. A significant reduction in gene expression of 77% was identified in family 2 compared to that in controls (*p*-value = 0.0038). A comparison of *FBN1* expression between the controls and the affected members from family 3 revealed a 50% decrease. For the affected members in families 4 and 5, a reduced gene expression by 43% and 85% was observed in the two families, respectively. A comparison of fibrillin-1 concentration between the controls and all the affected members from the five families shows that the expression is almost similar in both groups, with a slight decrease of 5.58% **(B)**. For individual families, the affected members in family 1 carrying the nonsense variant *FBN1*:c7180C>T exhibited fibrillin-1 levels almost equal to that of the control with a mild decrease of 2.5%. Similar findings were observed in family 2 with the frameshift variant *FBN1*:2142-2143delGC, with a 7% increase, while fibrillin-1 concentration in family 3 with the missense variant *FBN1*:c.209G>T was significantly reduced by 37.61% compared to that in the control (*p*-value = 0.0256). In families 4 and 5, fibrillin-1 levels were decreased by 8.4% and 45%, respectively.

Relative *FBN1*-mRNA levels in the blood samples from the 13 clinically affected family members were compared to those in 13 healthy controls (Figure 5A). Compared to the healthy controls, the relative gene expression of *FBN1* in all the affected patients was reduced by 50%. When comparing gene expressions among affected members within each family and controls, relative *FBN1*-mRNA expression in the affected members of family 1 was comparable to that in the controls, with a slight increase of 8%. Conversely, *FBN1*-mRNA expression in the affected members of family 2 was decreased by 77%. Affected members of family 3 exhibited a 50% reduction in *FBN1*-mRNA levels compared to those in the control. Although no genomic *FBN1* variants were identified in families 4 and 5, there was a reduction in *FBN1* expression by 56% and 85%, respectively.

### Evaluation of fibrillin-1 plasma levels using ELISA

The fibrillin-1 concentration in all the affected members was almost equal to that in the control, with a slight decrease of 5.58% (Figure 5B). Similar findings were obtained when comparing the protein concentration between the controls and affected members of families 1 and 2, with a slight increase of 2.5% and 7%, respectively. In family 3, the concentration of fibrillin-1 was reduced by 37.61% in the affected individuals compared to that in controls. In families 4 and 5, the protein concentration was decreased by 4% and 45%, respectively.

## Discussion

The *FBN1* gene, comprising 65 exons and encoding an 8,616 bp transcript and a 2,871 amino acid protein, is challenging to study due to its low expression in blood (FBN1 protein expression summary). Therefore, most studies investigating the *FBN1* sequence and expression are typically conducted using patient-derived fibroblasts, which hinders its implementation in routine clinical diagnostics (Tjeldhorn et al., 2015). To address this, we developed a cost-effective assay inspired by Miller et al. (2007) using two-round RT-PCR and Sanger sequencing to analyze the full *FBN1* transcript from the blood as the routinely tested sample type in the clinic. This assay costs USD 201.3, nearly half the USD 950 cost of ES, and it delivers results in 4 days–6 days compared to the 2 weeks–4 weeks required for ES. Its affordability, efficiency, and rapid turnaround make it a practical and non-invasive tool for detecting genetic variants and assessing their impacts. It offers a viable alternative to other methods that are often not covered by health insurance.

We successfully validated the designed assay in a cohort of five families with suspected MFS, and the findings for each family were unique, emphasizing the significance of the assay. In family 1, ES identified a heterozygous nonsense variant (*FBN1*:c.7180C>T), introducing a PTC 1,436 nucleotides upstream of the original stop codon. Typically, such nonsense pathogenic variants result in haploinsufficiency (HI), where the mutant transcript is degraded via nonsense-mediated decay (NMD), leaving only the WT allele to be expressed. This often leads to a milder phenotype (Kemezyte et al., 2023). However, RNA sequencing of the affected individuals revealed the presence of both the WT and mutant alleles, suggesting that the mutant transcript may have partially escaped NMD and was available for translation into a truncated protein. The presence of both alleles suggests a dominant negative (DN) effect, where the truncated protein interferes with the function of the WT protein, potentially explaining the severe cardiac manifestations observed in the affected family members. However, truncated FBN1 proteins that escape NMD may be nonfunctional, resulting in HI, which is also known to cause severe Marfan syndrome phenotypes, including early-onset cardiac manifestations. Arnaud et al. (2021) provided a detailed genotype–phenotype correlation, which demonstrated that *FBN1*-HI variants can result in a severe MFS phenotype. Therefore, functional analysis in disease-relevant tissues will be necessary to determine the actual pathogenic mechanism. Quantitative analysis showed that *FBN1-*mRNA levels in affected individuals were comparable to those in controls, supporting the expression of both alleles. However, since qPCR probes targeted a region common to both alleles, it was not possible to determine their relative contributions. Plasma fibrillin-1 levels in affected individuals were comparable to those in controls, suggesting that both alleles were actively transcribed and translated. This contrasts with other reported nonsense variants, such as *FBN1*: c.3217G>T, where only the WT allele was detected due to efficient degradation of the mutant transcript via NMD, resulting in a milder phenotype (Niu et al., 2020). Similarly, *FBN1*:c.6339T>A was associated with a 53% reduction in gene expression, which is also attributed to NMD-degradation (Tjeldhorn et al., 2015). These findings highlight that nonsense variants can lead to different transcriptional and phenotypic outcomes, depending on whether the mutant transcript is degraded via NMD or escapes surveillance and exerts a DN effect. In this case, the presence of both WT and mutant transcripts, along with comparable gene expression and protein levels, suggests that the truncated protein disrupts normal fibrillin-1 function, contributing to a more severe clinical phenotype than that typically observed in HI cases. This underscores the importance of RNA sequencing in distinguishing between HI and DN mechanisms in disease pathogenesis.

In family 2, ES identified a novel heterozygous frameshift variant (NM_000138.5(FBN1): c.2142_2143del (p.Pro715Argfs*8)) in exon 18 of the *FBN1* gene. This pathogenic variant is expected to produce a WT allele alongside a mutant transcript containing a PTC, which would undergo NMD (Sakai et al., 2016). RNA sequencing confirmed the exclusive expression of the mutant allele, with the WT allele being undetectable in all affected family members. Quantitative analysis showed a 77% reduction in overall *FBN1-*mRNA expression compared to that in controls, suggesting that most transcripts are degraded, with the residual expression primarily from the mutant allele. Plasma fibrillin-1 levels, measured via ELISA, were comparable to those in controls despite significantly reduced mRNA expression, implying a post-transcriptional regulatory mechanism stabilizing the protein to compensate for decreased gene expression. This phenomenon aligns with previous findings in other genes, such as *ACE2*, where high protein levels were maintained despite low mRNA levels (Wang et al., 2020), indicating a tightly regulated process of protein homeostasis.

The mutated transcript encodes a truncated protein lacking 2,148 amino acids, thus eliminating critical functional domains. This defective protein likely fails to integrate into the ECM, preventing proper LTBP binding and allowing excessive TGF-β signaling. This dysregulation contributes to the severe cardiac manifestations observed in affected individuals (Franken et al., 2015). Despite frameshift variants with PTC-mediated HI often being associated with milder phenotypes, the RNA sequencing and protein expression data suggest a dominant-negative effect in this case. The mutant fibrillin-1 protein appears deposited in the ECM, exacerbating disease severity. This explains the severe clinical phenotype in family 2, including aortic root dilation requiring surgical intervention and lens implantation.

A missense variant, c.209G>T, was identified in family 3, leading to the substitution of glycine with valine at position 70. Typically, heterozygous missense variants are associated with DN effects, where both WT and mutant alleles are expressed, and the mutant protein interferes with the WT protein (Chen et al., 2022). However, RNA sequencing of the c.209G>T variant in family 3 revealed exclusive expression of the mutated allele, with no detectable WT allele. This suggests that the WT allele is either transcriptionally silenced or degraded, leading to HI. This dual mechanism—the DN effect and HI—contributed to a 50% reduction in *FBN1-*mRNA levels, as confirmed by qPCR, and a 37.6% decrease in plasma fibrillin-1 protein levels, as determined by ELISA. Such findings would not have been detected through ES or predictive tools alone.

The c.209G>T variant affects the N-terminal domain of fibrillin-1, a region associated with ocular manifestations such as EL, which is observed in both affected patients. While high or normal *FBN1* levels in other missense pathogenic variants are often linked to severe phenotypes (Halliday et al., 1999; Whiteman and Handford, 2003), the reduced gene expression and protein levels may explain the mild-to-moderate cardiac involvement observed in these individuals.

RNA sequencing also highlighted the significance of transcript-level analysis. A previous study on the c.4925A>G variant in exon 39 initially predicted a p.Asp1642Gly missense change (Tjeldhorn et al., 2015). However, cDNA sequencing revealed a cryptic splice site activation, leading to an in-frame deletion of 18 nucleotides, a mechanism that is undetectable by DNA-based testing alone. Similarly, RNA sequencing of the *FBN1*:c.6354C>T synonymous variant demonstrated exclusive expression of the mutated allele due to disrupted splicing, leading to exon 51 deletion. A comparable effect was noted for the frameshift variant c.4905delC, with minimal WT expression. The exclusive expression of the mutated allele may be attributed to genetic imprinting, where one parental allele is preferentially expressed while the other is silenced (Miller et al., 2007). Additionally, MFS is increasingly recognized to follow a DN mechanism, where mutant transcripts exert a more substantial effect than the WT allele. A study by Dietz, 2022 demonstrated this phenomenon, showing that FBN1c.247 + 1G>A, a splice-site variant causing exon-two skipping, led to the exclusive expression of the mutant allele, significantly reducing fibrillin-1 deposition and disrupting microfibril architecture, even in the presence of two WT alleles (Eldadah et al., 1995). These findings emphasize the critical role of RNA sequencing in accurately characterizing variant effects beyond the capabilities of DNA sequencing alone.

In family 4, despite the proband exhibiting marfanoid features, ES failed to detect any pathogenic *FBN1* variants associated with MFS. However, RNA sequencing identified a novel pathogenic frameshift variant (NM_000138.5(FBN1):c.3945dup (p.Gly1316Argfs*10)), which was absent at the DNA level in both the exome and Sanger sequencing. This variant was confirmed through RNA sequencing of two separate blood samples from the proband, where cDNA synthesis and targeted amplification verified its presence. Forward and reverse sequencing of the proband’s RNA, alongside family and control samples, ruled out technical errors, suggesting that adenine (A) insertion was an RNA transcription error rather than a DNA mutation. Given that this insertion occurred in a poly-A stretch (AAAAAA), it is likely due to RNA polymerase slippage, where the polymerase loses its position and inserts extra nucleotides during transcription.

The concept of RNA polymerase slippage was first proposed by Chamberlin and Berg (1962) (Sakai et al., 2016) and later studied by Wagner et al. (1990), who demonstrated its role in restoring the reading frame of the lacZ construct in E.coli through slippage-induced nucleotide insertions. Similarly, Gordon et al. showed that transcriptional errors from slippage could increase phenotypic switching in bacterial systems. MacRae et al. reported a case of frameshift correction in the apolipoprotein B gene in humans, where an apoB86 truncated allele (caused by a single cytosine deletion) was corrected via RNA polymerase slippage, leading to the restoration of the full-length apoB100 protein (Linton et al., 1997). Based on these findings, we hypothesize that RNA polymerase slippage in *FBN1* caused the insertion of an extra A, leading to a pathogenic frameshift variant, the production of a mutant fibrillin-1 protein, and ultimately, the development of marfanoid features. This is the first report of RNA polymerase slippage contributing to MFS pathogenesis. Relative *FBN1-*mRNA levels were reduced, suggesting that the mutant transcript underwent NMD. Although some mutant transcripts were successfully degraded, others escaped NMD and contributed to disease manifestation.

In family 5, despite the proband exhibiting mild marfanoid features, ES did not identify any pathogenic *FBN1* variants. RNA sequencing further confirmed this finding, which ruled out any functional impact of non-coding variants detected by ES. The detection rate of *FBN1* mutations in individuals meeting the clinical criteria of MFS is estimated to be 93% (Stengl et al., 2020), which means that approximately 7% of the cases lack identifiable *FBN1* variants. However, such a percentage varies across studies (Stengl et al., 2020; Chen et al., 2022; Baetens et al., 2011).

Both relative *FBN1* expression levels and plasma fibrillin-1 concentrations in family 5 were notably reduced. This observation can be influenced by interacting partner proteins, genes, or transcription factors, such as the transcription factor PARP1 and Sp1 (Guo et al., 2013), or other mechanisms, including epigenetic regulation (Arai et al., 2020).

Clinically, the 2010 Ghent nosology is the standard criterion for MFS diagnosis. However, younger patients may have subtle or incomplete manifestations as many features—including cardiovascular involvement—may only appear or worsen with age (Salik and Rawla, 2025). Thus, the cases of the proband of family 4, who presented only with joint laxity, and family 5, where the proband had an increased height beyond the 95th percentile, raised the suspicion of MFS despite the patients not fulfilling the strict Ghent criteria.

## Conclusion

We underscore the necessity of performing an RNA-based sequencing approach for variant detection and functional analyses to validate the pathogenicity of exonic and intronic variants and advocate for revisiting standard diagnostic protocols in cases of clinically suspected or diagnosed MFS where genetic findings are either normal or classified as VUS. The ignorance of the mechanisms underlying the variable clinical expression and penetrance defects severely limits the potential of genetic counseling.

A limitation of our study is that NMD efficiency and RNA processing can vary across different tissue types. In addition, in the context of MFS, fibroblasts provide a more physiologically relevant context for assessing *FBN1* variant effects. Although our current study focused on blood-derived RNA for practical and accessibility reasons, we recognize the importance of validating these findings in patient-derived fibroblasts to determine whether the RNA-level changes observed in blood are similarly present in disease-relevant tissues. Future studies will aim to culture fibroblasts from patients carrying these variants to determine whether the RNA-level changes observed in blood are similarly present in fibroblast-derived RNA.

The small sample size, which is primarily due to the family-based nature of the investigation and the rarity of MFS, is another limitation. As a relatively uncommon hereditary connective tissue disorder, MFS presents significant challenges in recruiting large cohorts, especially within the framework of a study focused on detailed functional and transcriptomic analysis. The scope of our research required the inclusion of individuals from the same family to assess the segregation of findings, particularly the expression and functional impact of *FBN1* at the mRNA level. However, the availability of suitable and willing participants within affected families was inherently limited.

## Data Availability

The data that support the findings of this study are openly available in the Mendeley Data repository at the following DOI identifier: 10.17632/bcckk9r769.1.

## Supplementary material

SUPPLEMENTARY FIGURE S1 Gel electrophoresis of nested RT-PCR products used to amplify complete *FBN1*-mRNA. Lane 1 represents the RT-PCR product of the negative control; the reaction was applied using water instead of RNA and a primer that was blindly chosen from the 17 primers used in nested RT-PCR. Lanes A–Q represent the bands obtained by gel electrogenesis following RT-PCR amplification using the *FBN1*-mRNA-specific primers nested A–Q, respectively.

SUPPLEMENTARY FIGURE S2 Targeted screening for NM_000138.5(FBN1):c.3945dup (p.Gly1316Argfs*10). Screening results for the NM_000138.5(FBN1):c.3945dup (p.Gly1316Argfs*10) variant in the entire cohort using primer Nes-H revealed the insertion of an additional adenine exclusively in the proband of family 4 (IV:1).
