# Supplementary material for: A diagnostic RNA sequencing assay for direct identification and interpretation of pathogenic variants in the FBN1 gene
Source: Front Mol Biosci. 2025 Nov 3;12:1693943. doi: 10.3389/fmolb.2025.1693943 (PMC12620623; doi:10.3389/fmolb.2025.1693943)
Supplement: Supplementary file 1 [file Table1.docx]

Table S1. All *FBN1* variants identified in the probands of the five families by whole exome sequencing.

| **Family/**  **Proband**  **ID** | **FBN1**  **Variant** | **A.A Change** | **Allele** | **Variant ID** | **Consequence** | ***gnomAD**  **MAF** | ***ERG**  **MAF** | **ACMG**  **Classification** |
| --- | --- | --- | --- | --- | --- | --- | --- | --- |
| 1 (II:) | c.7571-113C>A | - | Heterozygous | rs1820488 | Intronic | 0.8802 | 0.9193 | Benign |
|  | c.7180C>T | p.Arg2394* | Heterozygous | rs397515848 | Stop gained | 0.0000006 | 0.000003 | Pathogenic |
|  | c.6496+128A>G | - | Heterozygous | rs363820 | Intronic | 0.1069 | 0.1805 | Benign |
|  | c.6314-64A>G | - | Heterozygous | rs2042746 | Intronic | 0.8837 | 0.9213 | Benign |
|  | c.5297-111G>A | - | Heterozygous | rs62011394 | Intronic | 0.0008951 | 0.0006 | VUS |
|  | c.5224+111A>G | - | Homozygous | rs9806595 | Intronic | 0.3255 | 0.2998 | Benign |
|  | c.4748-77T>G | - | Heterozygous | rs55694948 | Intronic | 0.1794 | 0.2177 | Benign |
|  | c.4337-55T>A | - | Heterozygous | rs75227249 | Intronic | 0.1103 | 0.0793 | Benign |
|  | c.4336+92delT | - | Heterozygous | rs398027180 | Intronic | 0.1545 | 0.0892 | Benign |
|  | c.3965-1962_3965-1961dupAA | - | Heterozygous | rs56969171 | Intronic | 0.03024 | 0.0782 | Benign |
|  | c.3965-1965_3965-1961delAAAAA | - | Heterozygous | rs1205382046 | Intronic | NA | NA | VUS |
|  | c.3965-2197G>A | - | Heterozygous | rs35026266 | Intronic | 0.2758 | 0.3032 | Benign |
|  | c.3589+134C>T | - | Heterozygous | rs12907671 | Intronic | 0.1720 | 0.1141 | Benign |
|  | c.3589+67_3589+71delGTTAT | - | Heterozygous | rs138563627 | Intronic | 0.2637 | 0.2956 | Benign |
|  | c.3589+36_3589+40delTTTTA | - | Heterozygous | rs72158035 | Intronic | 0.1752 | 0.1152 | Benign |
|  | c.3464-5G>A | - | Heterozygous | rs11853943 | Intronic | 0.1687 | 0.1153 | Benign |
|  | c.2729-132A>G | - | Heterozygous | rs34837775 | Intronic | 0.1209 | 0.0948 | Benign |
|  | c.2168-46A>G | - | Homozygous | rs140605 | Intronic | 0.2786 | 0.3196 | Benign |
|  | c.2113+107C>T | - | Homozygous | rs16961033 | Intronic | 0.2395 | 0.1285 | Benign |
|  | c.1960+98A>T | - | Heterozygous | rs35464791 | Intronic | 0.1206 | 0.09444 | Benign |
|  | c.1875T>C | p.Asn625= | Homozygous | rs25458 | Synonymous | 0.1925 | 0.1383 | Benign |
|  | c.1838-80G>T | - | Homozygous | rs8037304 | Intronic | 0.2387 | 0.1285 | Benign |
|  | c.1588+109C>T | - | Homozygous | rs2306352 | Intronic | 0.3698 | 0.3249 | Benign |
|  | **c.1415G>A** | p.Tyr472= | Homozygous | rs4775765 | Synonymous | 1 | NA | Benign |
|  | c.989-93C>A | - | Heterozygous | rs2413907 | Intronic | 0.1615 | 0.1107 | Benign |
|  | c.737-26delT | ­- | Heterozygous | rs373084568 | Intronic | 0.2447 | 0.0957 | Benign |
|  | c.248-103T>C | - | Homozygous | rs1018148 | Intronic | 0.9285 | 0.8286 | Benign |
| 2(II:9) | :c.2142_2143delGC | p.Pro715Argfs*8 | Heterozygous | - | Frameshift | NA | NA | Likely Pathogenic |
|  | **c.1415G>A** | p.Tyr472= | Heterozygous | [rs4775765](https://varsome.com/variant/hg19/rs4775765?&annotation-mode=germline) | Synonymous | 1 | NA | Benign |
| 3(III:1) | c.7571-113C>A | - | Heterozygous | rs1820488 | Intronic variant | 0.8802 | 0.9193 | Benign |
|  | c.6997+17C>G | - | Homozygous | [rs363832](http://www.ncbi.nlm.nih.gov/snp/rs363832) | Intronic | 0.746 | 0.6923 | Benign |
|  | c.6314-64A>G | - | Heterozygous | rs2042746 | Intronic | 0.8837 | 0.9213 | Benign |
|  | c.6037+54T>A | - | Homozygous | rs2303502 | Intronic | 0.6569 | 0.6797 | Benign |
|  | c.5297-85_5297-82dupACAC | - | Heterozygous | rs6145556 | Intronic | 0.5429 | 0.0015 | Benign |
|  | c.5297-111G>A | - | Heterozygous | rs62011394 | Intronic | 0.000895 | 0.0006 | VUS |
|  | c.4336+92delT | - | Heterozygous | rs398027180 | Intronic | 0.1545 | 0.0892 | Benign |
|  | c.1415G>A | p. Tyr472= | Homozygous | rs4775765 | Synonymous | 1 | NA | Benign |
|  | c.248-103T>C | - | Homozygous | rs1018148 | Intronic | 0.9285 | 0.82862 | Benign |
|  | c.209G>T | p. Gly70Val | Heterozygous | - | Missense | NA | 0.00001 | Likely Pathogenic |
| 4(IV:1) | c.*43A>T | - | Heterozygous | [rs144710695](http://www.ncbi.nlm.nih.gov/snp/rs144710695) | 3'-UTR | 0.000528 | 0.00069 | Likely Benign |
|  | c.7571-113C>A | - | Heterozygous | rs1820488 | Intronic variant | 0.8802 | 0.9193 | Benign |
|  | c.6997+17C>G | - | Homozygous | [rs363832](http://www.ncbi.nlm.nih.gov/snp/rs363832) | Intronic | 0.746 | 0.6923 | Benign |
|  | c.6314-64A>G | - | Heterozygous | rs2042746 | Intronic | 0.8837 | 0.9213 | Benign |
|  | c.6037+54T>A | - | Homozygous | rs2303502 | Intronic | 0.6569 | 0.6797 | Benign |
|  | c.5224+111A>G | - | Homozygous | rs9806595 | Intronic | 0.3255 | 0.2998 | Benign |
|  | c.4337-55T>A | - | Heterozygous | rs75227249 | Intronic | 0.1103 | 0.0793 | Benign |
|  | c.4336+92delT | - | Heterozygous | rs398027180 | Intronic | 0.1545 | 0.0892 | Benign |
|  | c.3965-2197G>A | - | Heterozygous | rs35026266 | Intronic | 0.2758 | 0.3032 | Benign |
|  | c.3589+134C>T | - | Heterozygous | rs12907671 | Intronic | 0.1720 | 0.1141 | Benign |
|  | c.3589+67_3589+71delGTTAT | - | Heterozygous | rs138563627 | Intronic | 0.2637 | 0.2956 | Benign |
|  | c.3589+36_3589+40delTTTTA | - | Heterozygous | rs72158035 | Intronic | 0.1752 | 0.1152 | Benign |
|  | c.3464-5G>A | - | Heterozygous | rs11853943 | Intronic | 0.1687 | 0.1153 | Benign |
|  | c.2729-132A>G | - | Heterozygous | rs34837775 | Intronic | 0.1209 | 0.0948 | Benign |
|  | c.2168-46A>G | - | Homozygous | rs140605 | Intronic | 0.2786 | 0.3196 | Benign |
|  | c.2113+107C>T | - | Homozygous | rs16961033 | Intronic | 0.2395 | 0.1285 | Benign |
|  | c.1960+98A>T | - | Heterozygous | rs35464791 | Intronic | 0.1206 | 0.0944 | Benign |
|  | c.1875T>C | p. Asn625= | Heterozygous | [rs25458](http://www.ncbi.nlm.nih.gov/snp/rs25458) | Synonymous | 0.1925 | 0.1383 | Benign |
|  | c.1838-80G>T | - | Homozygous | rs8037304 | Intronic | 0.2387 | 0.1285 | Benign |
|  | c.1588+109C>T | - | Homozygous | rs2306352 | Intronic | 0.3698 | 0.3249 | Benign |
|  | **c.1415G>A** | p. Tyr472= | Homozygous | rs4775765 | Synonymous | 1 | NA | Benign |
|  | c.989-93C>A | - | Heterozygous | rs2413907 | Intronic | 0.1615 | 0.1107 | Benign |
|  | c.737-26delT | ­- | Heterozygous | rs373084568 | Intronic | 0.2447 | 0.0957 | Benign |
|  | c.248-103T>C | - | Homozygous | rs1018148 | Intronic | 0.9285 | 0.8286 | Benign |
| 5(II:1) | c.248-103T>C | - | Homozygous | rs1018148 | Intronic | 0.9285 | 0.8286 | Benign |
|  | c.4336+92delT | - | Heterozygous | rs398027180 | Intronic | 0.1545 | 0.0892 | Benign |

*MAF: Minor Allele Frequency. *ERG: Emirati Reference Genome
