# Supplementary figures and images for: A diagnostic RNA sequencing assay for direct identification and interpretation of pathogenic variants in the FBN1 gene
Source: Front Mol Biosci. 2025 Nov 3;12:1693943. doi: 10.3389/fmolb.2025.1693943 (PMC12620623; doi:10.3389/fmolb.2025.1693943)

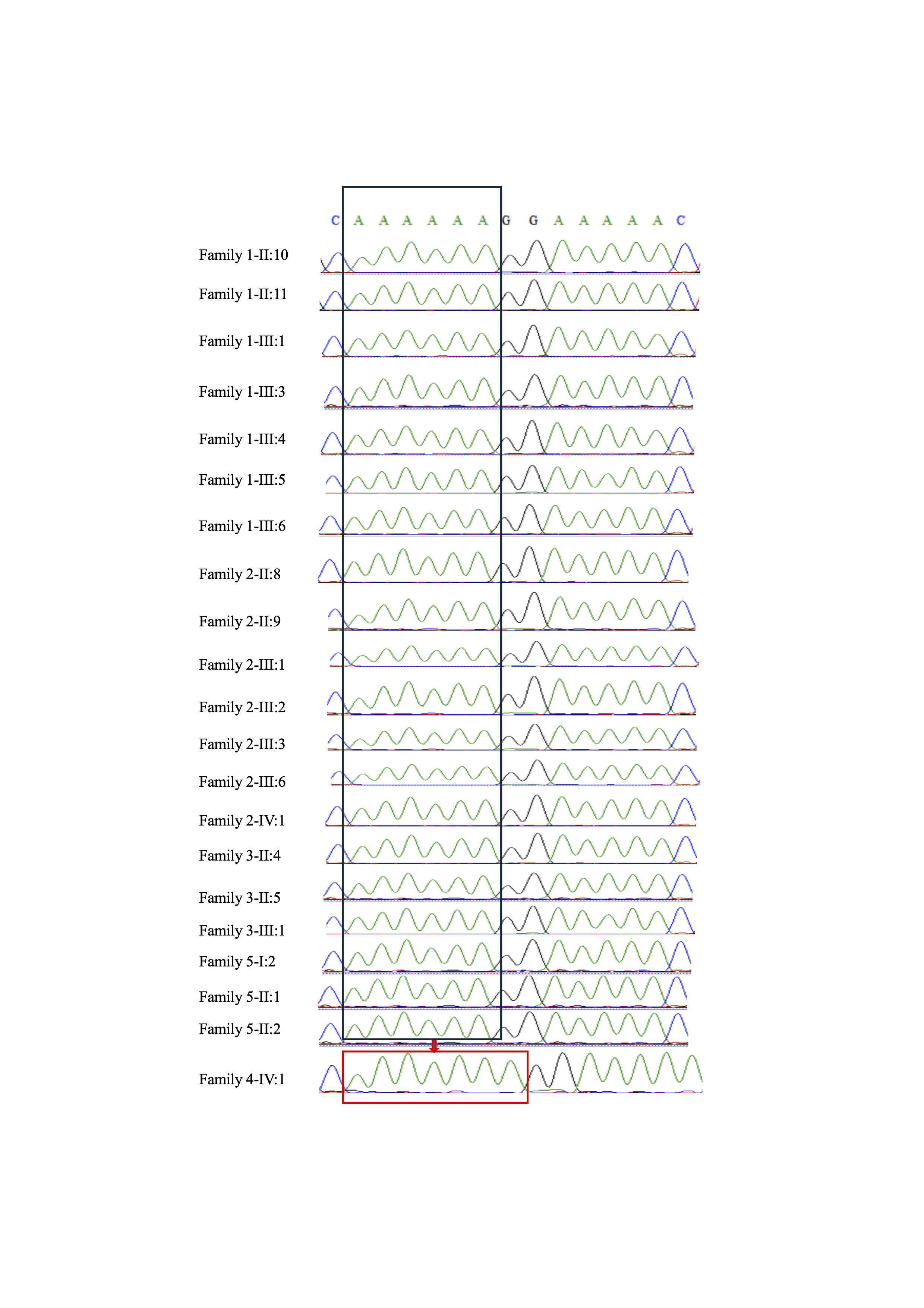

Supplement: Supplementary file 2 [file Image2.tif]

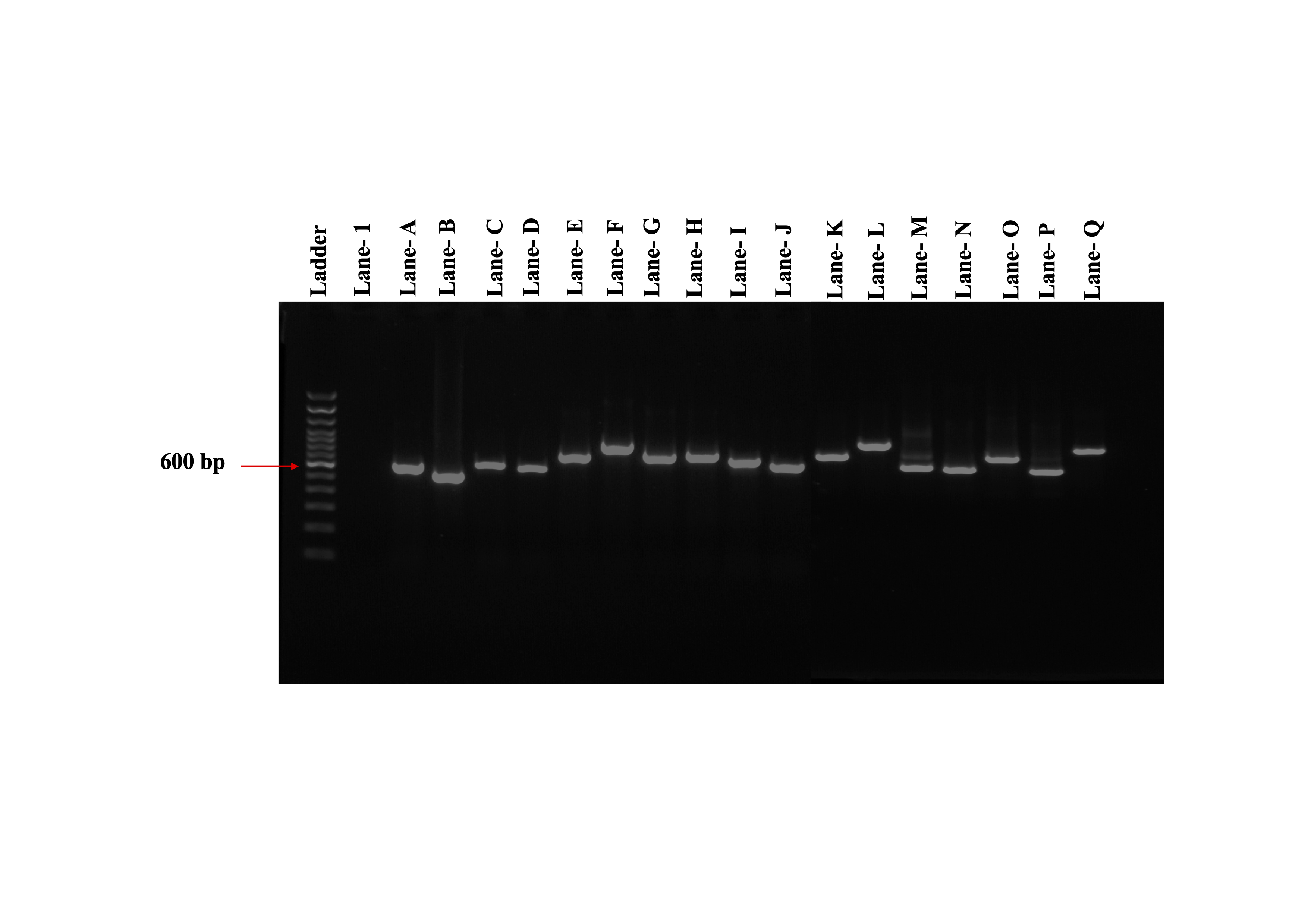

Supplement: Supplementary file 3 [file Image1.tif]
