# Supplementary material for: A diagnostic RNA sequencing assay for direct identification and interpretation of pathogenic variants in the FBN1 gene
Source: Front Mol Biosci. 2025 Nov 3;12:1693943. doi: 10.3389/fmolb.2025.1693943 (PMC12620623; doi:10.3389/fmolb.2025.1693943)
Supplement: Supplementary file 4 [file Table2.docx]

Table S2. The candidate disease causing variants identified in the probands by ES, and the primers and PCR conditions utilized to amplify the DNA segment harboring the identified variants.

| Family/  Pedigree ID | The target variants | Forward Primer | Reverse Primer | Amplicon size | Initial denaturation | Denaturation | *Tm | Extension | Final extension |
| --- | --- | --- | --- | --- | --- | --- | --- | --- | --- |
| 1/II:11 | *FBN1*: c.7180C>T | TGTCTCCAGCTTTCCCCTCT | GGTCCTAAGCACACACAGCT | 568 | 95°c/  2min | 95°c/ 30sec | 58°c/30 sec | 72°c/30 sec | 72°c/7min |
| 2/III:1 | *FBN1*:c.2142_2143delGC | ATGTGTGCAAAACCAAGGG | AATCCTAGAGCCCACAGTGC | 323 | 95°c/  2min | 95°c/ 30sec | 60°c/30 sec | 72°c/30 sec | 72°c/7min |
| 3/III:1 | *FBN1*:c.209G>T | ACCAACCCAGCATTGAGTCT | AGCAGCAATAATACCCTTTCATTCA | 537 | 95°c/  2min | 95°c/ 30sec | 58°c/30 sec | 72°c/30 sec | 72°c/7min |
| 4/IV:1 | *FBN2*: c.2308A>C | CTCTTTGAATTTCTGCCAGCGT | TGTTTGATTCTGATATTTTCCTGTCA | **525** | 95°c/  2min | 95°c/ 30sec | 58°c/30 sec | 72°c/30 sec | 72°c/7min |
| 5/II:1 | *MEIS2*:c.245+15delC | TCTTTTTCCCTTTCCACCCCC | GGCTAGTTCTTCGGGGCTTT | 506 | 95°c/  2min | 95°c/ 30sec | 60°c/30 sec | 72°c/30 sec | 72°c/7min |

*Tm: Annealing Temperature
